# Pregnancy and Accelerated Phase of Myeloid Chronic Leukemia Treated with Imatinib: A Case Report from a Developing Country

**DOI:** 10.1155/2016/6104948

**Published:** 2016-03-31

**Authors:** Lydie Ocini Ngolet, Innocent Kocko, Alexis Elira Dokekias

**Affiliations:** Department of Medical Hematology, Brazzaville Teaching Hospital, 13 Avenue Auxence Ikonga, BP 32, Brazzaville, Congo

## Abstract

*Background*. Chronic myeloid leukemia is a hematological malignancy caused by expression of BCR-ABL tyrosine kinase oncogene, product of the t(9;22) Philadelphia translocation. Accelerated phase of this disease marks the onset of advanced rapidly progressive disease unresponsive to many therapies. Pregnancy limits broad number of therapies on patients because of their potential teratogenic effects. We report the case of a pregnant 34-year-old patient on accelerated phase successively managed by imatinib. She achieved a safe pregnancy and delivered at 39 weeks a healthy baby without congenital abnormalities. Our case is unusual because of the accelerated phase of the disease.* Case Presentation*. A 34-year-old African female with history of chronic phase of myeloid leukemia on imatinib, lost to follow-up for 4 months, presented to the hematological department for abdominal discomfort. Accelerated phase of chronic myeloid leukemia was diagnosed. Complete hematological response was achieved on high doses of imatinib. At the completion of 39 weeks, she delivered a healthy child without congenital anomalies.* Conclusion*. Despite its teratogenic and embryotoxic effects, front line imatinib is the only effective, well-tolerated treatment for patient on accelerated phase that can be offered to patients in sub-Saharan countries.

## 1. Background

Chronic myeloid leukemia (CML) is a clonal myeloproliferative disorder characterized by Philadelphia chromosome that results from a reciprocal translocation between chromosome 9 and chromosome 22. This disease may follow biphasic or triphasic course that is usually diagnosed at chronic phase (CP). The natural history of CML on patients under imatinib had changed significantly since the majority of the patients are not progressing beyond CP. In our context, accelerated phase (AP) is usually diagnosed on CP CML patients who progressed to AP because they discontinued imatinib.

Accelerated phase is defined by either at least 10–19% of blasts in peripheral blood or bone marrow cells or at least 20% peripheral basophils or thrombocytopenia less than 100 G/L unrelated to therapy or increasing spleen size and increasing white blood cell count unresponsive to therapy and genetic and karyotypic abnormalities.

Imatinib is available free of charge in the Congo since 2005 through nongovernmental organization. It is the first-line treatment for CP CML. It had changed the natural history of chronic myeloid leukemia by showing overall survival benefits. CML affects in Africa mostly young men and women in reproductive age with desire of pregnancy [1, 2]. We report the case of an uneventful pregnancy that was successively managed with imatinib on a patient with CML that progressed from CP to AP.

## 2. Case Presentation

A 34-year-old African female patient was admitted at the Hematology Department of Brazzaville Teaching Hospital for investigation of malignant leukocytosis on June 2014. She was asymptomatic and presented at the initial examination with an enlarged spleen palpable at 17 cm below the left costal margin. Blood count showed a hyperleukocytosis at 298 G/L with the following distribution: 1% blast, 2% promyelocytes, 4% myelocytes, 9% metamyelocytes, 73% neutrophils, 6% eosinophils, 2% monocytes, and 3% lymphocytes. Hemoglobin rate was 10.9 g/dL and platelet was 489 G/L. Cytogenetic studies performed on bone marrow aspiration showed positive Philadelphia chromosome.

Based on clinical, hematological, and cytogenetic analyses, high risk CML at chronic phase was diagnosed. Complete hematological response was achieved on imatinib 400 mg per day combined with hydroxyurea 4 g per day in 21 days.

Cytogenetic response was studied in bone marrow aspiration 6 months after imatinib initiation in December 2014. It showed partial cytogenetic response.

On imatinib during 11 months and regular monitoring, the patient discontinued imatinib and was lost to follow-up during 4 months. She presented 4 months later at the outpatient clinic in August 2015 pregnant (12 weeks) with recurrence of an enlarged spleen at 10 cm below the left costal margin and leukocytosis. WBC count was 57 G/L with 10% myeloblasts, 7% promyelocytes, 9% myelocytes, 6% metamyelocytes, 52% neutrophils, 10% eosinophils, 2% monocytes, and 3% lymphocytes. Hemoglobin rate was 9.5 g/dL and platelet was 189 G/L. Bone marrow cytogenetic studies did not show additional chromosomic translocation.

Based on the rate of the blast in peripheral blood AP CML was diagnosed because the blast rate in the peripheral blood was at 10%. Second complete hematological response was obtained on imatinib 600 mg per day and return to CP was observed in 30 days. Imatinib dose was then reduced at 400 mg per day. She remained on that dose during all her pregnancy.

At the completion of 39 weeks of gestation, she delivered a female healthy child weighing 2.87 Kg with Apgar scores 8/10 and 10/10 at 1 and 5 minutes without any congenital abnormalities.

The patient has regular monitoring by hemogram every two months and she was supplied. The infant attended well-child visit at 1 and 2 months old. She is healthy and in good condition and is now 2 and a half months old. Abdominal and kidney ultrasound did not show any malformations.

## 3. Discussion

Incidence of pregnancy associated with CML is low and no consensus exists on how to manage pregnancy situation in CML [3, 4]. Many case reports and report series showed the successful outcome of patients with pregnancies that were presenting CML at chronic phase on imatinib [4–6]. One of the largest studies evaluated 180 pregnancies of patients receiving imatinib for CML. Outcome data were available for 125 patients. Among them 28% underwent elective terminations, 14% had a miscarriage, and 12 cases of fetal abnormalities were reported [6].

Our patient discontinued the imatinib during four months. She presented at the hospital with a pregnancy of 12 weeks and recurrence of malignant leukocytosis. There was a concern about the progression of the disease, resistance to imatinib, and the pregnancy. Therapies as interferon *α* and leukapheresis are of safer use during the pregnancy but are not available in the Congo [7, 8]. In light of normal fetal ultrasound and because termination of pregnancy is not acceptable in our sociocultural environment, we decided, despite the limited data on the safety of imatinib in pregnancy, its teratogenic effects on rats reported, to manage the patient with imatinib [4–6].

Challenges to manage this patient were multiple. First, introducing imatinib at the first trimester of pregnancy is controversial. A number of cases of complications that included spontaneous abortion, malformation, and low birth weight were reported [9], while other studies described satisfactory outcomes of pregnancies under normal dose of imatinib [4–6]. Second, we have to manage the patient with high dose of imatinib. Data outcomes of pregnancies under high dose of imatinib are unknown. High dose of imatinib introduced in first trimester may increase rate of complications.

Our patient was presenting CML at accelerated phase while she was pregnant. This is another particularity of our report while most case reports and series involved pregnancies and CML at chronic phase [4–6, 10]. Hydroxyurea could not be used to reduce the leukocytosis since it inhibits DNA synthesis and have potential to cause abortion, intrauterine growth retardation, and congenial malformation [11].

Even though uses of contraceptive methods are recommended to our patients under chemotherapy, we do have to face sociocultural factors in our countries which encourage women to give birth.

## 4. Conclusion

This case report and other report series suggest that patients with CML can keep receiving imatinib during their pregnancies.
